# PP65 The Use Of The Procalcitonin Test As A Diagnostic Marker For Neonatal Sepsis: Systematic Review

**DOI:** 10.1017/S0266462325102845

**Published:** 2025-12-29

**Authors:** Dandiany Camily Kuczera Sofka, Daniela Gorski, Caroline Mensor Folchini, Yeo Jim Kinoshita Moon, Fernanda Carolina Cruz Evangelista, Mariana Millan Fachi

## Abstract

**Introduction:**

Neonatal sepsis remains a major cause of morbidity and mortality in neonatology. While blood culture is considered the gold standard for detecting sepsis, its low sensitivity significantly limits its utility. The identification of biomarkers, such as procalcitonin (PCT), is essential for improving diagnostic accuracy. This Scientific Technical Opinion critically evaluated the available evidence on the role of PCT in diagnosing neonatal sepsis and clinical practice.

**Methods:**

This study followed Joanna Briggs institute and Cochrane Collaboration guidelines and was registered with PROSPERO. Systematic searches were conducted in Embase and MEDLINE. Eligible studies included randomized, quasi-randomized, cohort, case-control, observational, and cross-sectional designs that evaluated PCT diagnostic accuracy for neonatal early or late sepsis, microbiologically or clinically documented. Studies had to perform testing at the onset of sepsis symptoms, prior to antimicrobial therapy. Data collected included true positive, true negative, false positive, and false negative results. Sensitivity and specificity meta-analyses were performed, and summary receiver operating characteristic curves were generated using Meta-DiSc 2.0 software, with results reported as 95 percent confidence intervals (CIs).

**Results:**

A total of 111 studies were analyzed, involving 19,175 neonates. The sensitivity of PCT in diagnosing sepsis was 0.787 (95% CI: 0.757, 0.815) and the specificity was 0.853 (95% CI: 0.822, 0.879). In the “time of collection” group analysis, we obtained the following results: at zero hours, sensitivity was 0.753 (95% CI: 0.695, 0.802) and specificity was 0.803 (95% CI: 0.739, 0.854); up to 12 hours, sensitivity was 0.912 (95% CI: 0.748, 0.973) and specificity was 0.827 (95% CI: 0.643, 0.927).

**Conclusions:**

The findings suggested that PCT demonstrates high diagnostic accuracy for neonatal sepsis. Thus, incorporating PCT could enhance diagnostic precision and help minimize the unnecessary use of antimicrobials, ultimately supporting better outcomes in antimicrobial stewardship programs within neonatal intensive care units.

